# Supplementary material for: Lyoprotective Effects of Mannitol and Lactose Compared to Sucrose and Trehalose: Sildenafil Citrate Liposomes as a Case Study
Source: Pharmaceutics. 2021 Jul 28;13(8):1164. doi: 10.3390/pharmaceutics13081164 (PMC8400243; doi:10.3390/pharmaceutics13081164)
Supplement: Supplementary file 1 [file pharmaceutics-13-01164-s001.zip › pharmaceutics-1254994-supplementary.pdf]

pharmaceutics MDPI

Supplementary Materials: Lyoprotective Effects of Mannitol and Lactose Compared to Sucrose and Trehalose: Sildenafil Citrate Liposomes as a Case Study

María José de Jesús Valle, Andreía Alves, Paula Coutinho, Maximiano Prata Ribeiro, Cristina Maderuelo and Amparo Sánchez Navarro

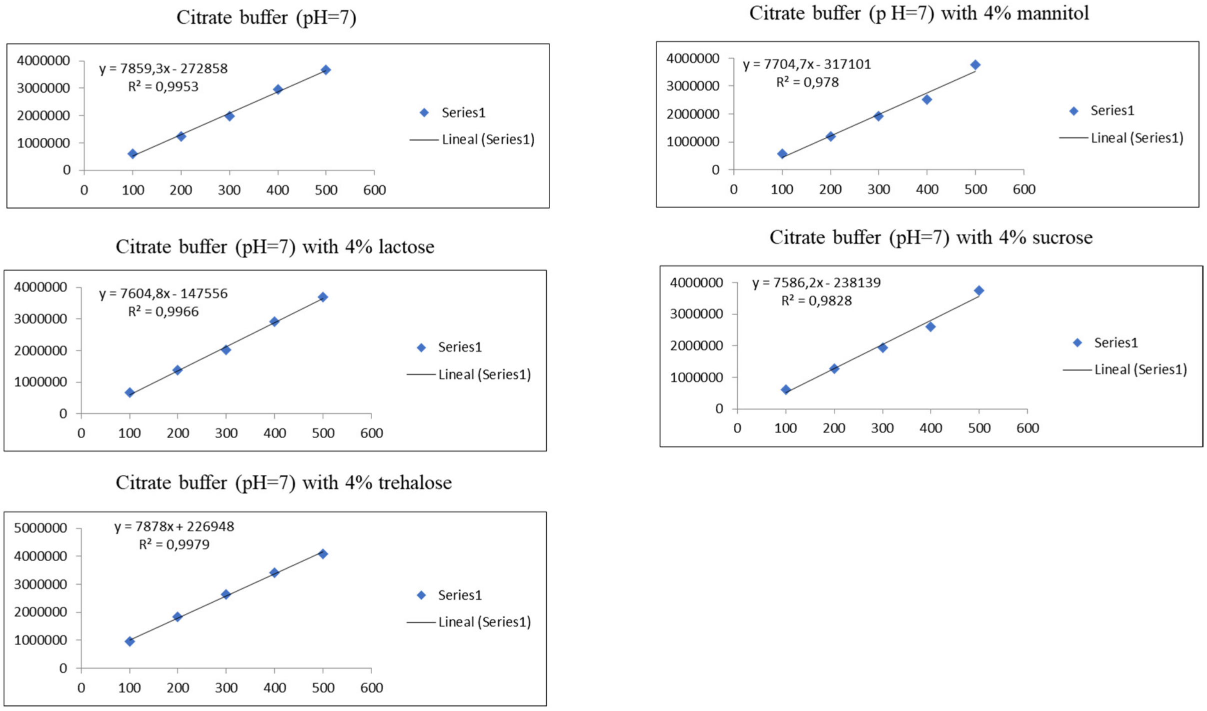

Figure S1. Linear relationship between chromatographic peak area (y) and drug concentration (x) for sildenafil citrate standard samples with and without additive. (p = 0.4060 for the slope comparison).

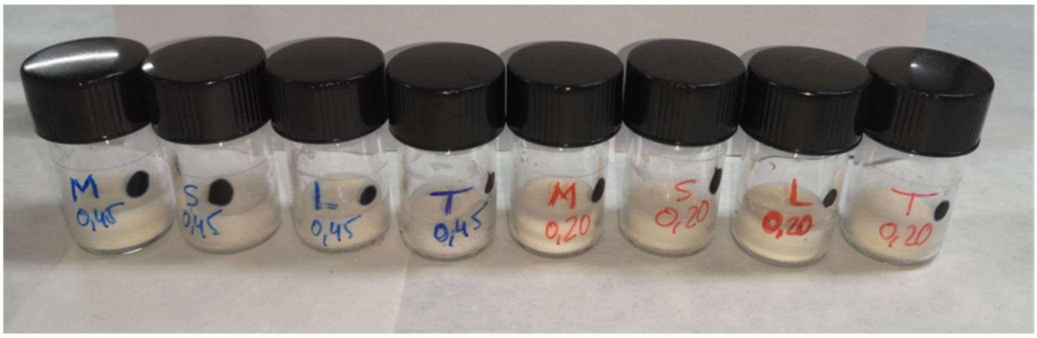

Figure S2. Aspect of cakes obtained after liophylisation of small and large liposomes with manitol (M), lactose (L), sucrose (S) or trehalose (T).

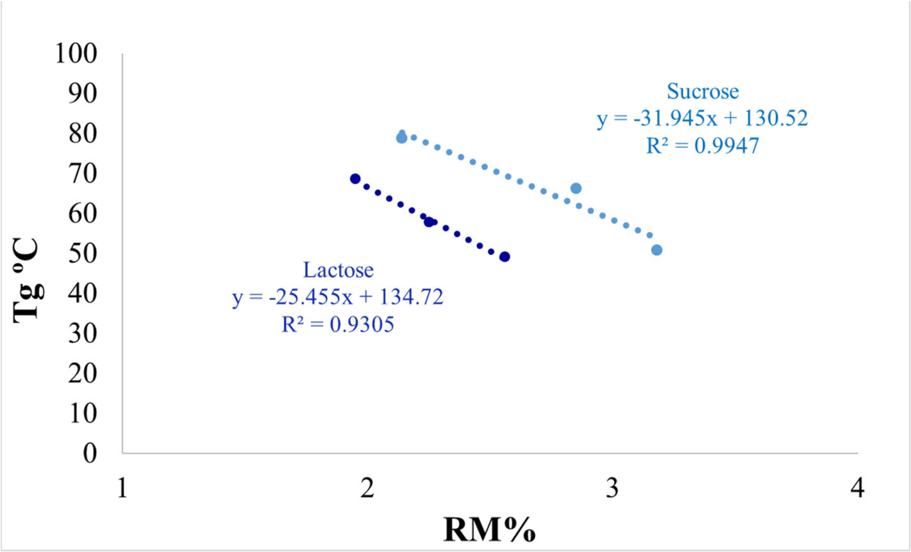

Figure S3. Linear relationship between remaining moisture (RM) and the onset of the peak associated to Tg.

Table S1. Thermal analysis of lyophilised samples with different additive.

Additive	Residual moisture (%)	Onset (ºC)	Endset (ºC)	Tª peak (ºC)	Integral (mJ)	dH (J/g)
**Mannitol**	3.01	63.50	75.60	71.04	63.75	-12.88
**Mannitol**	2.57	65.20	76.82	72.04	-63.15	-12.53
**Mannitol**	2.56	58.73	74.44	70.19	-46.32	-11.41
**Lactose**	3.18	50.92	67.49	59.22	-7.52	-1.53
**Lactose**	2.85	66.35	110.95	77.85	-54.08	-11.58
**Lactose**	2.14	78.92	106.29	87.87	-44.05	-8.57
**Sucrose**	2.56	49.14	57.03	53.38	-2.03	-0.46
**Sucrose**	2.25	57.82	67.52	62.37	-3.82	-0.85
**Sucrose**	1.95	68.64	84.15	75.55	-14.09	-2.76
**Threalose**	2.53	88.67	105.17	97.90	-7.58	-1.43
**Threalose**	2.35	54.12	85.98	75.10	-11.06	-2.19
**Threalose**	2.07	45.82	90.90	62.60	-28.87	-6.40
